# High-Performance Self-Powered UV Detector Based on SnO_2_-TiO_2_ Nanomace Arrays

**DOI:** 10.1186/s11671-018-2501-x

**Published:** 2018-04-03

**Authors:** Duo Chen, Lin Wei, Lingpan Meng, Dong Wang, Yanxue Chen, Yufeng Tian, Shishen Yan, Liangmo Mei, Jun Jiao

**Affiliations:** 1 School of Physics and State Key Laboratory of Crystal Materials, Shandong University, Jinan, 250100 People’s Republic of China; 2 School of Microelectronics, Shandong University, Jinan, 250100 People’s Republic of China; 3 Department of Mechanical and Materials Engineering, Portland State University, P.O. Box 751, Portland, OR 97207-0751 USA

**Keywords:** Self-powered, UV detectors, SnO_2_-TiO_2_ nanomace arrays, Heterojunction

## Abstract

Photoelectrochemical cell-typed self-powered UV detectors have attracted intensive research interest due to their low cost, simple fabrication process, and fast response. In this paper, SnO_2_-TiO_2_ nanomace arrays composed of SnO_2_ nanotube trunk and TiO_2_ nanobranches were prepared using soft chemical methods, and an environment-friendly self-powered UV photodetector using this nanostructure as the photoanode was assembled. Due to the synergistic effect of greatly accelerated electron-hole separation, enhanced surface area, and reduced charge recombination provided by SnO_2_-TiO_2_ nanomace array, the nanostructured detector displays an excellent performance over that based on bare SnO_2_ arrays. The impact of the growing time of TiO_2_ branches on the performance of UV photodetector was systematically studied. The device based on optimized SnO_2_-TiO_2_ nanomace arrays exhibits a high responsivity of 0.145 A/W at 365 nm, a fast rising time of 0.037 s, and a decay time of 0.015 s, as well as excellent spectral selectivity. This self-powered photodetector is a promising candidate for high-sensitivity, high-speed UV-detecting application.

## Background

Ultraviolet photodetectors (UVPDs) have been widely used in many fields, such as remote control, chemical analysis, water purification, flame detection, early missile plume detection, and secure space-to-space communication [1]. To avoid the use of costly UV pass filters and achieve visible-blind operation, wide bandgap semiconductors have been studied widely for light detecting, especially in the ultraviolet region [2]. In recent decades, nanostructured semiconductors such as nanorods, nanowires, nanotubes, and nanobranches have attracted extensive research interest due to their high surface-to-volume ratio and rationally designed surface morphology [3–13]. Photoelectrochemical cell (PEC)-typed photodetectors assembled with nanostructured semiconductors exhibit a high responsivity and a fast transient response compared with traditional photoconductive semiconductor thin film detectors. As a new and efficient way to fabricate high-performance photodetectors, PEC-based devices can avoid complicated epitaxial processes and expensive single crystal substrates, which is very important for the growing cheaper optoelectronic applications. Therefore, self-powered UVPDs based on PEC device have attracted intensive research interest. Self-powered UVPDs based on PEC structure have been fabricated using a liquid I^−^/I_3_^−^ redox couple electrolyte [14–18] and a nanocrystalline TiO_2_ film [14] or a multilayer TiO_2_ nanorod-assembled cloth/nanorod array-based electrode [15]. Impressive performances were observed in these UVPDs. However, liquid I^−^/I_3_^−^ redox couple electrolyte is not ideal for long-term operation: it is highly corrosive, volatile, and photoreactive, interacting with common metallic components and sealing materials. From this point, water-based electrolytes may be the most safe, most stable, and most environment-friendly electrolyte. Zhang et al. have reported a UV-visible photodetector based on ZnO/CuO heterojunctions and NaSO_4_ aqueous solution, which shows an excellent photodetection performance [19]. TiO_2_ has attracted a great deal of attention due to its outstanding physical and chemical properties for water electrolyte-based UVPDs. Lee et al. reported a UV detector based on a TiO_2_ film/water solid–liquid heterojunction [20], which exhibits high photosensitivity, excellent spectral selectivity and fast response. In order to further enlarge the TiO_2_/electrolyte contact area, Xie et al. fabricated a self-powered PEC photodetector based on TiO_2_ nanorod arrays/water UVPD [21]. Until now, water electrolyte-based UVPDs still show a lower photoresponsivity than those using I^−^/I_3_^−^ redox couple electrolyte. Moreover, low electron mobility of TiO_2_ increases the probability of photon-induced electron recombination with the electrolyte. By contrast, SnO_2_ possesses a high electron mobility, suggesting a faster diffusion transport of photon-induced electrons to the transparent conductive oxide current collector. Recently, high-quality TiO_2_/SnO_2_ heterojunction nanostructures have been prepared by different methods for optoelectronic applications [17, 22]. Impressive performance has been observed in UVPDs using TiO_2_/SnO_2_ branched heterojunctions and SnO_2_ mesoporous spheres @ TiO_2_ as electrode materials [16, 17]. However, all these UVPDs were assembled with disordered nanostructures. It can be expected that if ordered SnO_2_-TiO_2_ nanostructure arrays with a high electron transport efficiency are adopted as the photoanode of the UVPDs, a much better photodetecting performance can be obtained.

**Fig. 1:**
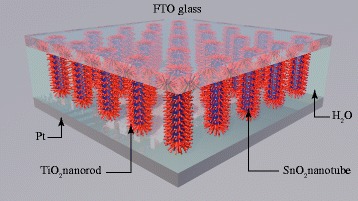
The schematic structure of the nanostructured SnO_2_-TiO_2_/H_2_O solid-liquid heterojunction-based UV detector

In this work, ordered SnO_2_-TiO_2_ nanomace arrays (STNMAs) was synthesized using soft chemical methods. An environment-friendly self-powered UVPD was assembled using the STNMAs as photoanode and water as electrolyte. The schematic structure of STNMAs/H_2_O UVPD is shown in Fig. 1. STNMAs grown vertically on fluorine-doped tin oxide (FTO) glass were used as the active electrode. The STNMA-based device shows a higher photocurrent density than that of the bare SnO_2_ nanotube-based device under UV irradiation. The spectral photosensitivity and response time are characterized to evaluate the potential of the STNMA UVPD. The impact of the growing time of TiO_2_ branches on the performance of UV photodetector was also studied. The self-powered UVPD based on optimized STNMAs exhibits a high responsivity of 0.145 A/W, a fast rise time of 0.037 s, and a decay time of 0.015 s, as well as excellent spectral selectivity. Moreover, the electrolyte of this photodetector is water, which is low cost, stable, and environment friendly.

## Methods

### Synthesis of SnO_2_ Nanotube Arrays

FTO glass (2 cm × 2 cm) was ultrasonically cleaned with ethanol and deionized water for 15 min respectively and then dried in the air. A 10-nm Sn film was deposited on FTO by thermal evaporation and annealed in air at 550 °C for 1 h to form a dense SnO_2_ layer. High-quality ZnO nanorod arrays were prepared on the SnO_2_ buffered FTO glass by a two-step hydrothermal method. The details could be found in our previous work [23]. SnO_2_ shell layer was deposited on the ZnO nanorod array by a liquid phase deposition. FTO covered with ZnO nanorod arrays was immersed in Na_2_SnO_3_ aqueous solution at 60 °C for 1 h. Then the sample was immersed in 0.01 M dilute hydrochloric acid to remove the ZnO template, and uniform SnO_2_ nanotube arrays (SNAs) were obtained.

### Synthesis of SnO_2_-TiO_2_ Nanomace Arrays

TiO_2_ nanobranches were grown on the SnO_2_ nanotube trunk by a simple aqueous chemical growth method. The SnO_2_ nanotube arrays on FTO glass prepared above were put in an aqueous solution of 0.2 M TiCl_4_ at room temperature. In order to achieve different TiO_2_ nanobranch length, the deposition was conducted at 6, 12, 18, and 24 h respectively. The resulted STNMAs were thoroughly rinsed with deionized water and then annealed at 450 °C for 30 min.

### Assemble of the UV Detector

The PEC-typed photodetector was assembled in a similar structure of a dye-sensitized solar cell, as discussed in our previous work [24]. In brief, the obtained STNMAs synthesized on FTO glass were used as the active electrode and a 20-nm-thick Pt film deposited on FTO glass by magnetron sputtering is adopted as the counter electrode. The active electrode (SnO_2_/FTO) and the counter electrode (Pt/FTO) were adhered together face to face with a 60-μm-thick sealing material (SX-1170-60, Solaronix SA, Aubonne, Switzerland). Finally, deionized water was injected into the space between the top and counter electrodes. The effective area of the UV detector was approximately 0.2 cm^2^.

### Characterization

The crystal structure of the samples was examined by X-ray diffraction (XRD; XD-3, PG Instruments Ltd., Beijing, China) with Cu Kα radiation (*λ* = 0.154 nm). The surface morphology of the samples was characterized using a field emission scanning electron microscope (FESEM; Hitachi S-4800, Hitachi, Ltd., Chiyoda, Tokyo, Japan) and a transmission electron microscope (TEM; F-20, FEI Company, Hillsboro, OR, USA). The optical transmittance was measured using an UV-visible dual beam spectrophotometer (TU-1900, PG Instruments, Ltd., Beijing, China). A 500-W Xenon lamp (7ILX500, 7Star Optical Instruments Co., Beijing, China) with a monochromator (7ISW30, 7Star Optical Instruments Co.) was used as UV light source to generate monochromatic light for the spectral response characterization. The spectral photoresponse characteristics were obtained by a programmable sourcemeter (2400, Keithley Instruments Inc., Cleveland, OH, USA). The photoresponse switching behavior measurement was obtained by an electrochemical workstation (RST5200, Zhengzhou Shirusi Instrument Technology Co. Ltd., Zhengzhou, China).

## Results and Discussion

**Fig. 2:**
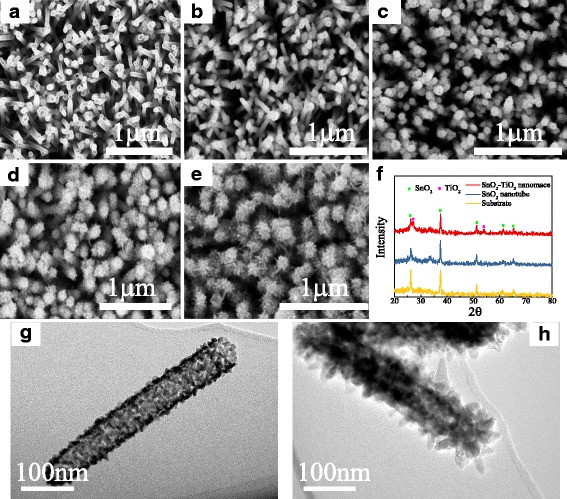
SEM and TEM images and XRD patterns of SnO_2_ nanotube arrays and SnO_2_-TiO_2_ nanomace arrays. **a** High-magnification top-view SEM image of SnO_2_ nanotube arrays. **b** SEM image of 6-h-grown STNMAs. **c** SEM image of 12-h-grown STNMAs. **d** SEM image of 18-h-grown STNMAs. **e** SEM image of 24-h-grown STNMAs. **f** X-ray diffraction patterns of the substrate, SnO_2_ nanotube arrays, and STNMAs. **g** TEM image of bare SNA. **h** TEM image of 18-h-grown STNMAs

Morphology of SnO_2_ nanotube arrays (SNAs) and STNMAs was examined by a FESEM. As shown in Fig. 2a, ordered SNAs with opened top were grown uniformly on the surface of FTO glass substrate. Further analysis indicates that the nanotubes have a diameter of 50–80 nm and a wall thickness less than 10 nm. The density of nanotubes is typically 30 nanotube/μm^2^. Figure 2b–e illustrates the SnO_2_ nanotube arrays immersed in TiCl_4_ solution for 6, 12, 18, and 24 h, respectively. It can be clearly seen that the SnO_2_ nanotubes grow almost vertically to the FTO substrate and are covered with a large number of TiO_2_ nanobranches to form a nanomace structure. The morphology of SNA and STNMA is also checked by TEM. As shown in Fig. 2g, h for the bare SNA and STNMA grown for 18 h, the SnO_2_ nanotube has a length of about 500 nm and the TiO2 branches grow tightly on the wall of SnO_2_ nanotubes. The morphology of the STNMAs is strongly dependent on the growth time. As the growth time increased, the branches become more numerous and longer. These nanobranches coated on SnO_2_ nanotube would greatly enlarge the specific surface area and roughness, which is important for PEC applications. However, once the deposition time reaches 24 h or longer, the branches will form continuous network that greatly suppresses the effective active area, which would result in a decrease of the active area of TiO_2_ contacted with the electrolyte. This is confirmed by the reduced performance of the photodetector in the following part. The crystal structure of the SNAs and STNMAs with 18 h deposition time was examined by X-ray diffraction (XRD), and the corresponding patterns are presented in Fig. 2f. The 2*θ* scan pattern shows that all the peaks of the SnO_2_ nanotubes is consistent with those of the FTO substrate, which can be indexed to SnO_2_ rutile structure [JCPDS No. 77-0450.]. After the deposition of TiO_2_ nanobranches, two more peaks appear, corresponding to the (110) and (211) planes of the rutile TiO_2_ [JCPDS No. 02-0494.]. The XRD results indicate that the STNMAs are composed of rutile SnO_2_ nanotube trunk and rutile TiO_2_ nanobranches without other phases.

**Fig. 3:**
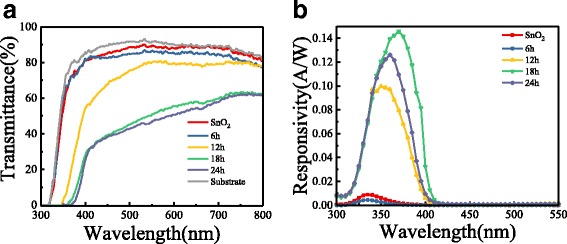
The UV-visible transmittance spectra and responsivity spectrum of photodetectors. **a** Spectrum of transmittance for FTO glass substrate, SNAs, and STNMAs with different growth time. **b** Responsivity spectrum of photodetectors based on SNAs and STNMAs

The transmission spectrum of the FTO glass, SNAs, and STNMAs is shown in Fig. 3a. A sharp absorption edge located at 320 nm can be observed for FTO glass. The absorption edge of SnO_2_ nanotube arrays and 6-h-grown STNMAs is similar to that of the FTO glass, but the absorption edge of 12–24-h-grown STNMAs all show an obvious red shift. The transmittance of FTO reaches to zero when the wavelength is shorter than 305 nm, which determines the spectral response edge in the short-wavelength region. The strong light scattering by the TiO_2_ nanobranches causes a lower transmittance of all STNMAs than that of FTO and SnO_2_ nanotubes in the wavelength range of 400–550 nm. From these transmittance spectra, it can be concluded that only light with the wavelength between 305 and 400 nm can be well absorbed by TiO_2_ arrays and contribute to the UV photoresponsivity, which is confirmed in the following spectral response characterization. The spectral responsivity of these photodetectors was measured in the range of 300–550 nm at zero bias, as shown in Fig. 3b. The responsivity is calculated by the following formula: *R* = *I*/*AE*, where *R* is the responsivity, *I* is the measured photocurrent, *A* is the active area of the photodetector device, and *E* is the irradiance intensity of the light source, which is measured by a standard light power meter. The device performs as a self-powered photodetector that operates at a nominal zero-applied voltage, with a large photocurrent response under a weak light illumination. As shown in Fig. 3b, the maximum responsivity value for a bare SNA-based UV photodetector is approximately 0.01 A/W at 335 nm, corresponding to an incident photon-to-current conversion efficiency (IPCE) of only 3.7%. Normally, oxygen vacancy can be easily formed in SnO_2_ material and cause a high charge recombination. TiO_2_ nanobranch deposition on the SNAs can passivate the surface of SnO_2_ and reduce the electron-hole recombination. The STNMA-based photodetectors show much better UV photoresponsivity. The peak responsivity of STNMAs grown for 18 h is approximately 0.145 A/W at 365 nm. The corresponding IPCE is higher than 49.2%, which is much higher than other H_2_O-based PEC detectors at this wavelength [20, 23, 24]. Considering the loss of incident photons caused by the light absorption and scattering of the FTO glass, much higher internal quantum efficiency can be expected. The TiO_2_ nanobranches coated on the SnO_2_ nanotube arrays greatly increase not only the contact interface area between the STNMAs and the electrolyte but also the light scattering ability, resulting in an enhancement of the photon harvesting efficiency. Furthermore, these ultrathin branches are very effective at transporting holes to the TiO_2_/water interface as most electron-hole pairs are formed within the diffusion length, ultimately minimizing the recombination loss. Moreover, the photoelectrons injected into SnO_2_ nanotube from TiO_2_ nanobranch reach the FTO collecting electrode rapidly because SnO_2_ possesses a higher electron mobility than TiO_2_. When the growth time reaches 24 h or longer, the branches on the nanotube arrays are interconnected. The active area of TiO_2_ contacted with the electrolyte decreases. Therefore, an excessively long growth time is disadvantageous and leads to a reduced photovoltaic performance of the UV photodetector.

**Fig. 4:**
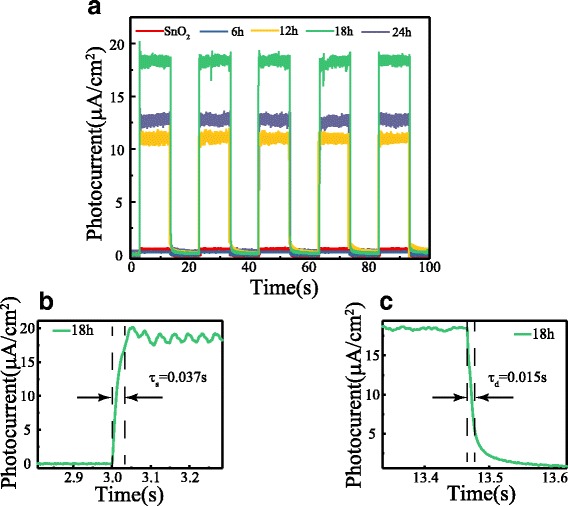
Time response of the STNMAs/water UV detector. **a** Photocurrent response under on/off radiation of 129 μW/cm^2^ UV light illumination. **b** Enlarged rising and **c** decaying edge of the photocurrent response

To characterize their responsivity to a fast-changing light signal, the photocurrent density-time characteristics of the devices were measured at 0 V bias under an intermittent 365 nm UV-light irradiation with a power of 129 μW/cm^2^. The incident radiation is switched with an on/off interval of 10 s. Five repeated cycles are displayed in Fig. 4a, which indicates that the photocurrent can be reproducibly switched between the “ON” state and the “OFF” state by periodically turning the UV light on and off. When the deposition time of TiO_2_ nanobranch is less than 6 h, the photocurrent density is quite low. In this case, only TiO_2_ nanoparticles with a high defect density were formed on the surface of SnO_2_ nanotube, which would result in a high electron-hole recombination and a poor photoresponse. With the increase of growth time, the crystal quality of the TiO_2_ nanobranches was improved and the surface area was greatly increased. Therefore, the photocurrent has a significant increase when the growth time is longer than 6 h and reaches the maximum when the deposition time is 18 h. From the enlarged rising and decaying edges of the photocurrent response curve, the rising time and decay time of the UV detector are approximately 0.037 and 0.015 s (Fig. 4b, c), indicating a rapid photoresponse characteristic. The quantitative criterion for the rising time is the time to reach 90% of the stable photocurrent, and that for decay time is time to reach 1/e (37%) of the original photocurrent. The overall performance of the STNMA-based self-powered UV detector is considerably better than that reported by other works, as compared in Table 1.

**Table 1:** Comparison of the self-powered UVPDs with other works

Electrode	Electrolyte	Wavelength of peak (nm)	Responsivity (A/W)	Efficiency (%)	Rising time (s)	Decay time (s)	Reference
SnO_2_ mesoporous spheres@TiO_2_	I^−^/I_3_^−^	350	0.113	42.6	0.007	0.006	[16]
SnO_2_ nanotube-TiO_2_	I^−^/I_3_^−^	350	–	20	–	–	[25]
TiO_2_ nanorod arrays	H_2_O	365	0.025	8.4	0.15	0.05	[20]
ZnO nanoneedle arrays	H_2_O	385	0.022	7.1	0.1	0.1	[24]
ZnO nanorod-ZnS arrays	H_2_O	340	0.056	20.4	0.02	0.04	[23]
SnO_2_-TiO_2_ nanomace arrays	H_2_O	365	0.145	49.2	0.037	0.015	This work

**Fig. 5:**
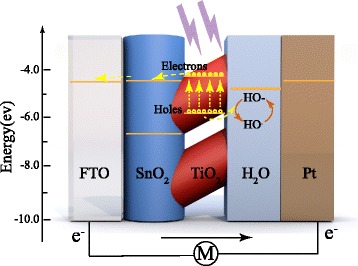
Schematic energy band diagram and the electron-transfer processes for the STNMAs/H_2_O heterojunction

Schematic diagram of energy band matching and device working mechanism are shown in Fig. 5. When the incident light travels through FTO glass and reaches the active layer of TiO_2_ nanobranches, photons with energy exceeding the TiO_2_ bandgap will be absorbed and electrons are excited from the valance band to the conduction band, and electron-hole pairs will be generated thereafter. The built-in potential across the interface works as the driving force to separate the electron-hole pairs. Negative electrons move along from TiO_2_ nanobranch to the SnO_2_ nanotube and get collected by the FTO electrode. These electrons will easily transfer into the external circuit and return to the Pt layer of the counter electrode since the work function of FTO matches with the conduction band of SnO_2_ and TiO_2_. The positive holes are driven to the surface of the TiO_2_ nanobranch and get captured by OH^−^ anion, the reduced form of the redox molecule (h^+^+OH^−^ → OH·). Fast removal of holes can be expected across the heterojunction due to the large surface area. The oxidized form of the redox molecule is reduced back to the reduced form OH^−^ at the counter electrode (Pt/FTO) by the electrons that re-entered into the UV detector from the external circuit (e^−^ + OH· → OH^−^). Here the Pt serves as both a catalyst for the redox reaction and conducting road for the electrons. The circuit was completed in this manner, demonstrating a self-powered UV detection property.

## Conclusions

In summary, we have synthesized SnO_2_-TiO_2_ nanomace arrays composed of SnO_2_ nanotube trunk and TiO_2_ nanobranches using soft chemical methods. A self-powered UV detector was assembled using this nanostructure as the active electrode and water as the electrolyte. Due to the accelerated electron-hole separation speed by the SnO_2_-TiO_2_ core-shell structure, enlarged surface area of TiO_2_ nanobranches, and fast electron transport property of SnO_2_ nanotube, an excellent performance was obtained in this nanostructured photodetector. For the detector based on the optical STNMAs, a high IPCE up to 49.2% is observed at 365 nm, which is more than 10 times larger than the maximum IPCE of bare SnO_2_ nanotube (3.7%). A rapid response time and an excellent spectral selectivity were also obtained in this photodetector. We believe that this SnO_2_-TiO_2_ nanomace structure can be extended to other applications based on photoelectrochemical effect, such as dye-sensitized solar cells and photoelectrochemical hydrogen production.

## Abbreviations

FTO: Fluorine-doped tin oxide
IPCE: Incident photon-to-current conversion efficiency
PEC: Photoelectrochemical cell
SEM: Scanning electron microscope
SNAs: SnO_2_ nanotube arrays
STNMAs: SnO_2_-TiO_2_ nanomace arrays
TEM: Transmission electron microscope
UV: Ultraviolet
UVPDs: Ultraviolet photodetectors
XRD: X-ray diffraction
